# Enzyme immobilization with plant-based polysaccharides through complex coacervation

**DOI:** 10.1016/j.lwt.2025.117537

**Published:** 2025-03-01

**Authors:** Waritsara Khongkomolsakul, Eunhye Yang, Younas Dadmohammadi, Hongmin Dong, Tiantian Lin, Yunan Huang, Alireza Abbaspourrad

**Affiliations:** Department of Food Science, College of Agriculture and Life Sciences, Cornell University, 243 Stocking Hall, Ithaca, NY, USA

**Keywords:** Enzyme immobilization, Phytase, Polysaccharide, Molecular docking, Protein-polysaccharide intermolecular interaction

## Abstract

Plant-based polysaccharides (PSs) were used to immobilize phytase in a coacervate system. Molecular docking predicted the intermolecular interactions and conformations between the phytase and the polysaccharide and correlated them to the activity recovery of phytase in the coacervate complex. PSs with two different functional groups, sulfate (iota (IC), lambda (LC), and kappa (KC) carrageenan) and carboxylate (low methoxyl pectin (LMP) and sodium alginate (SA)) were investigated. The optimized conditions for coacervation and activity recovery were pH 4 with a phytase-to-polysaccharide volume ratio of 12:1. Zeta potential measurements, FTIR spectroscopy, and molecular docking confirmed that electrostatic interactions and hydrogen bonding were the main driving forces for coacervate formation. Coacervate complexes of phytase formed with LMP, SA, or KC showed a high activity retention after immobilization, with approximately 30% yield of complex and 75% immobilization efficiency of the phytase. The lower enzyme activity retention observed for IC and LC complexes is attributed to these PSs binding to the enzyme's active site. Overall, this work contributes to the body of knowledge about intermolecular interactions between phytase and polysaccharides and can serve as a guide to formulating stable, functional ingredients for a plant-based diet.

## Highlights

• ●Molecular docking revealed that binding conformation can affect enzyme activity.
• Coacervate complex's optimums were pH 4 and 12:1 v/v phytase to polysaccharides.
• Optimum complex showed high encapsulation efficiency with high activity recovery.

## Graphical abstract

**Figure:**
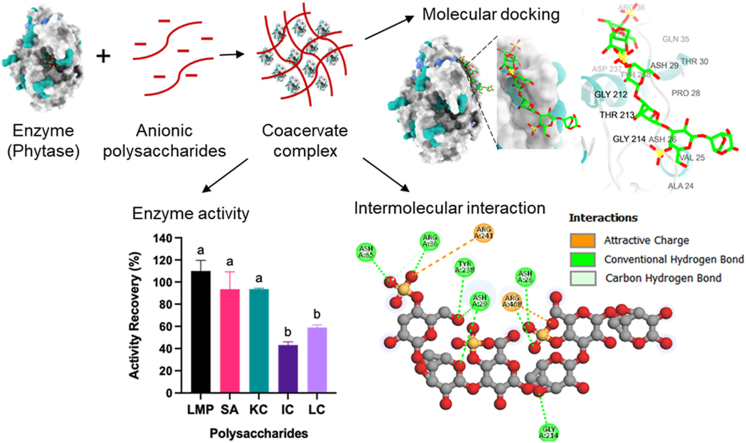

## 1 Introduction

Cereal, nuts, legumes, rice, and other plant seeds are typical in diets in low and medium-income countries (Sandstead & Freeland-Graves, 2014). These grains contain phytate (Phytic acid or Myo-inositol hexakisphosphate), and the seeds serve as the primary phosphorus source. Although phosphorus is an essential dietary nutrient, some phosphorus-containing molecules, like phytate, can also act as antinutrients by chelating positively charged ions such as Fe, Zn, and Ca and facilitating their removal from the digestive tract (Petroski & Minich, 2020). When these minerals are bound by phytate, they become insoluble, and their bioavailability decreases, exacerbating nutritional mineral deficiencies (Zimmermann & Hurrell, 2007).

Phytase, a phosphatase enzyme that hydrolyzes phytate into myo-inositol and phosphates, has gained recognition as a supplement in animal feed to enhance phosphorus content and to increase the bioavailability of the nutrients locked into phytic acid molecules within plant seeds (Greiner & Konietzny, 2006). In humans, several studies have shown that oral intake of phytase improves iron absorption with high phytate content in foods such as maize porridge (Nielsen et al., 2013). Nevertheless, integrating phytase into animal or human food has some crucial limitations.

Enzymes exhibit optimal activity within a specific range of pH and temperature and lose their stability under extreme conditions, such as acidic pH and high temperature (de Oliveira Carvalho & Orlanda, 2017). Commercially available phytases have been shown to have a maximum optimal temperature ranging from 37 to 70 °C and an optimal pH range of 3.0–5.5, based on the phytase source (Menezes-Blackburn et al., 2015; Rao et al., 2009). Integrating phytase in animal or human diets requires food processing such as pelleting and cooking, which usually involve high temperatures ranging from 85 to 90 °C for 1 min for pelleting applications and heating at 100 °C for 12–30 min during cooking. This food processing can denature the enzyme and inhibit the enzyme activity (Rao et al., 2009). Many companies have engineered enzymes that are more stable under thermal and gastric conditions; however, the expression and purification of these enzymes after modification are costly (Menezes-Blackburn et al., 2015). In addition, using genetically modified organisms (GMOs) could also impact consumer perspectives on the food product. Therefore, a techno-economically feasible and large-scale friendly platform is sought.

Several studies have tried to stabilize enzymes by using systems like protein immobilization to enhance the stability of enzymes through various carriers and systems, including crosslinking, hydrogel beads, electrospinning, and spray drying (Filippovich et al., 2023; Lin et al., 2023; E. Yang et al., 2024). However, most of the immobilizing methods, including crosslinking, altered the protein structure and decreased enzyme activity by 65%, illustrating the importance of choosing an immobilizing system that will not alter the enzyme structure.

Complex coacervation has emerged as a promising method with multiple applications, including protecting enzyme activity from external stresses, targeting enzyme delivery, and retaining enzymatic activity after enzyme immobilization (Maghraby et al., 2023; Perry & McClements, 2020; Wan et al., 2023). The formation of complex coacervates using the phase separation of oppositely charged food-grade polymers has been shown to have immobilization efficiencies as high as 99%, with high loading capacity and at a reasonable cost (Lin et al., 2022; Timilsena et al., 2019). Overall, complex coacervation enhances the stability of the enzyme and is much easier to scale up than electrospinning or hydrogel beads.

Plant-based biopolymers and polysaccharides are often nontoxic, biocompatible, cost-effective, and can functionally protect cargo ingredients (Eghbal et al., 2016; Kühtreiber et al., 1999). Low methoxyl pectin (LMP) and sodium alginate (SA) are anionic polysaccharides with carboxyl groups, which have been shown to enhance the immobilization efficiency of a protein while creating a smaller particle size and increasing protein stability (Mala & Anal, 2021; Niu et al., 2019; Weng et al., 2022). Carrageenans, such as kappa-carrageenan (KC), iota carrageenan (IC), and lambda carrageenan (LC), extracted from red seaweeds, exhibit similar interactions with alginate and have diverse functionalities attributed to their specific structural variations, with LC having the highest negative charge due to its three sulfate groups (Zhang et al., 2024).

Coacervates have been shown to form between biomolecules and biopolymers that are oppositely charged. By controlling the pH of a solution, the charge on the biopolymers can be adjusted and if the isoelectric points are sufficiently different, that is, on either side of neutral, electrostatics become a driving force for coacervation. The negative charges from either carboxyl or sulfate functional groups are a good choice as an immobilizer as they can form coacervates with positively charged proteins and enzymes (Abka-khajouei et al., 2022). We plan to exploit the inherent natural charge on our plant-based polysaccharides to make intermolecular associations to immobilized phytase and enhance its stability to heat and acidity (Wan et al., 2023).

It is still being determined how different polysaccharides affect enzyme coacervation because most studies only choose one type of polysaccharide to explore the enzyme's stability after complex formation. Thus, we chose to examine and elucidate the intermolecular interactions between five polysaccharides and phytase using molecular docking via AutoDock Vina as a stepping stone to form a more stable immobilized enzyme for food application and explore the impact of polysaccharides on enzyme stability and activity. The five anionic PSs were then divided into two groups based on their predominant functional groups. The carboxylate group was represented by low methoxyl pectin (LMP) and sodium alginate (SA), while the sulfate group included three kinds of carrageenans. We formulated and optimized the coacervate complex specifically for high-activity recovery after complex formation. All coacervates were characterized by zeta potential, particle size, turbidity, FTIR, SDS PAGE, and SEM. We hypothesize that the use of docking methods will provide a foundation for predicting the best combinations of polysaccharides and phytase and that the coacervates formed will stabilize the phytase with minimal enzymatic deactivation.

## 2 Materials and methods

### 2.1 Materials

κ-Carrageenan (KC), *ι*-carrageenan (IC), *λ*-carrageenan (LC) were provided by TIC GUMS (98%, Westchester, IL, USA). Low methoxyl pectin (GENU® pectin type LM 12 CG-Z 35% DE, LMP) was provided by CP Kelco (Lille Skensved, Denmark). Phytase from wheat (>0.01 unit mg^−1^ solid)(Phy), sodium alginate (medium viscosity, MW 200 kDa), sodium acetate, ammonium molybdate tetrahydrate, trichloroacetic acid (TCA), acetic acid glacial, and L-ascorbic acid (all reagent grade) were purchased from Sigma-Aldrich (St. Louis, MO, USA). Sodium phytate was purchased from Astatech (95%, Bristol, PA, USA). Sulfuric acid (95–98 %) was purchased from Fluka Honeywell (Charlotte, NC, USA). Hydrochloric acid, sodium hydroxide, and the Pierce™ Rapid Gold BCA protein assay kit were purchased from Thermo Fisher Scientific (Rockford, IL, USA). SDS-PAGE reagents, Mini-PROTEAN® Tetra hand-cast systems, and TGX FastCast acrylamide kit were purchased from BIO-RAD (Hercules, CA, USA). Milli-Q water (18.2 MΩ/cm) was purified using Millipore water (Millipore Sigma, Burlington, MA, USA).

### 2.2 Preparation of coacervate complex

Enzyme solution (Phytase, Phy) of 1 g/100 mL and all polysaccharide solutions of 1 g/100 mL were prepared separately by dissolving 100 mg of powder in 10 mL Milli-Q water and mixing on a rotator at 24 rpm overnight at room temperature (∼25 °C) for full hydration. The solutions were adjusted to pH 4 with 1 M NaOH and 1 M HCl to change the surface charge of each complex for coacervation. Phy and each polysaccharide solution were mixed at different ratios, and the pH was readjusted to 4. The complex was collected by centrifugation at 10,000*g* for 10 min at 4 °C. The complex pellets were frozen at −20 °C overnight or at least 12 h and put in the freeze drier for at least 24 h (Labcono, Kansas, MO, USA). The supernatants were collected for protein quantification using a BCA (bicinchoninic acid) protein assay kit to calculate the immobilization efficiency.

### 2.3 Complex optimization

#### 2.3.1 Turbidity analysis

The turbidity was used in order to confirm the complex formation in the solution. The turbidity value was obtained using the transmittance value of the complex mixtures (2 mg mL^−1^). The transmittance value was measured in a 1-cm path-length quartz cuvette using a UV–Vis spectrophotometer (UV-2600, SHIMADZU Co., Japan) at 600 nm according to (Lin et al., 2022). Transmittance value could be used to calculate the turbidity as follows:

(Eq.1) $$Turbidity=-\ln\frac{I}{I_{0}}$$

where *I* is the transmittance intensity of complex solution, and *I*_*0*_ is the transmittance intensity of quartz cuvette with the same volume of dispersed solution (Milli-Q water).

#### 2.3.2 Particle size and zeta-potential analyses

The particle size was measured to ensure the complex formation by comparing the size before and after the complexation of each ratio, while zeta potential was used to indicate the pH and ratio showing the strongest electrostatic interaction. The complex solutions' average particle size and zeta potential were measured using the Zetasizer Nano-ZS (Nano S, Malvern Instruments, Worcestershire, UK). Particle size measurements used a 1-cm path-length cuvette at a backscattering angle of 173° and 25 °C, while zeta potential used a folded capillary zeta cell at 150 V. The measurements were done in triplicates with 15 runs for each analysis. Other parameters were the same as those previously reported (Lin et al., 2022).

### 2.4 Immobilization efficiency and yield of phytase in complex

To quantify phytase concentration for immobilization efficiency, supernatants were analyzed using a Pierce™ Rapid Gold BCA (bicinchoninic acid) protein assay kit compared to the standard curve from phytase and BSA. A phytase standard curve was used to measure the concentration released from the complex, while a BSA standard curve was available in the supplement for comparison purposes (Fig. S1). Briefly, 20 μL of sample solution was placed into a 96-well plate and mixed with 200 μL working solution containing 50:1 of proprietary copper chelator and cupric sulfate, respectively. The absorbance was measured at 480 nm after 5 min of incubation using a plate reader (SpectraMax iD3, Molecular Devices, CA, US). The immobilization efficiency was calculated as the following equation:

(Eq.2) $$Immobilization\;efficiency\;\left(\%\right)=\frac{Total\;added\;phytase-Free\;phytase\;in\;supernatant}{Total\;added\;phytase}\times 100$$

The yield percentage of the complex was calculated from freeze-dried mass and theoretical solid content in the initial mixed solution, which can be calculated as the following equation:

(Eq.3) $$Yield\;\left(\%\right)=\frac{Mass\;of\;freeze\;dried\;complex\;}{Mass\;of\;total\;solid\;in\;the\;initial\;solution}\times 100$$

Loading capacity was measured to determine how much protein is in the complex powder in a weight-by-weight percentage. We obtained a mass of phytase from protein concentration in a redispersed freeze-dried powder at a concentration of 2 mg mL^−1^ in Milli-Q water after vortex, which was measured using Pierce™ Rapid Gold BCA (bicinchoninic acid) protein assay kit. A mass of freeze-dried complex was obtained from the amount that was redispersed. To calculate for loading capacity, we could use the following equation:

(Eq.4) $$Loading\;Capacity\;\left(\%\right)=\frac{Mass\;of\;enzyme\;in\;complex\;}{Mass\;of\;freeze\;dried\;complex}\times 100$$

### 2.5 Phytase activity assay

Phytase activity was measured using a molybdenum blue reaction to determine the amount of phosphate, a by-product of phytase hydrolysis on phytate, with an adjustment from a previous method (Duru Kamaci & Peksel, 2021; Nagul et al., 2015; E. Yang et al., 2024). A redispersed complex in water 150 μL was aliquoted into 350 μL sodium acetate buffer (AB) combined with 100 μL of substrate phytate (0.44 mM) dissolved in AB. The mixed solution was then incubated at 50 °C for 30 min, and the reaction was stopped by denaturing enzymes with trichloroacetic acid (TCA, 15 g/100 mL). The solution was then centrifuged and collected 100 μL of supernatant. The collected supernatant was pipetted into 900 μL of distilled water and 1 mL of color reagent consisting of ascorbic acid (10 g/100 mL), ammonium molybdate (2.5 g/100 mL), and 1 M sulfuric acid in the volume ratio of 1:1:3, respectively. The mixed solution was further incubated for 15 min at 50 °C and measured for the absorbance at 820 nm (A_1_). For the activity control (A_0_), follow the above protocol by adding TCA before the 30-min incubation at 50 °C. The final activity indicated by absorbance was then calculated using delta A (A_1_ - A_0_). Then, the delta A was compared to the standard curve of free phytase activity at different concentrations (Fig. S3(b)) to get a phytase concentration in the complex based on enzyme activity. The % activity recovery can be calculated as follows (Eq. (5)):

(Eq.5) $$Activity\;recovery\;\left(\%\right)=\frac{Activity\;of\;phytase\;in\;complex}{Activity\;of\;free\;phytase}\times 100$$

### 2.6 Complex characterization

#### 2.6.1 Fourier transform infrared spectroscopy (FTIR)

The freeze-dried powder samples were measured using an IR-Affinity-1 Spectrometer from Shimadzu Corporation (Kyoto, JP) to assess their functional group and intermolecular interaction within the complex and each polymer. The FTIR spectra were obtained using an average of 32 scans in duplicate from wavelength of 400–4000 cm^−1^ with a resolution of 4 cm^−1^.

#### 2.6.2 Sodium dodecyl sulfate-polyacrylamide gel electrophoresis (SDS-PAGE)

The coacervate complexes redispersed in Milli-Q water were analyzed using SDS-PAGE to determine protein molecular weight and quantity. TGX FastCast acrylamide kit was used to prepare the polyacrylamide gel. The protein sample was prepared by adding 25 μL of protein (2 mg mL^−1^) with 25 μL of 2X Laemmli sample buffer and incubated in a 50 °C water bath for 2 h. The gel was loaded with 20 μL of protein in the sample buffer. The loaded gel was then assembled and run on Mini-PROTEAN® Tetra hand-cast systems. The 1X running buffer was freshly made with 25 mM Tris (3.02 g Tris base), 0.192 M glycine (14.4 g/L glycine), and 1 g/L sodium dodecyl sulfate. The electrophoresis was performed at a constant of 110 V for about 1 h. The gel was stained using a staining buffer consisting of Coomassie B R-250, methanol, acetic acid, and Milli-Q water for 30 min and destained with destaining buffer consisting of methanol, acetic acid, and Milli-Q water overnight (Lin et al., 2022).

#### 2.6.3 Scanning electron microscope (SEM)

Freeze-dried powder samples were observed with a Zeiss Gemini 500 Scanning Electron Microscope (SEM) (Jena, DE). The powder sample was scattered on the stub with carbon tape. It was then coated with carbon using a Denton Desk V sputter coater (NJ, US). The images were captured with an accelerating voltage of 3 kV (EHT) and 20.0 μm aperture using a HE-SE2 detector (backscattered signal).

#### 2.6.4 Molecular docking

Molecular docking was used to predict the intermolecular interactions between the amino acids of phytase and the polysaccharides for further comparison with the enzyme activity of the coacervate complex. The phytase structure was retrieved from the Protein Data Bank (PDB code: 6GJ2) (Faba-Rodriguez et al., 2022). The phytase structure was obtained from the same source of origin, wheat, similar to the phytase that was used in this study. 6GJ2 was obtained through x-ray diffraction with a molecular weight of 61.52 kDa. In our coacervate system, the pH of the environment was adjusted to pH 4 before complexation; therefore, to perform molecular docking, we used the Adaptive Poisson–Boltzmann Solver (APBS) web service to get an appropriate protonated state of phytase using Amber force field (Amber FF) at pH 7 and adjust to pH 4 using CHARMM-GUI (http://www.charmm-gui.org/) (Jo et al., 2008, 2014; Jurrus et al., 2018; Park et al., 2023). The protonation state of amino acids adjusted with Amber FF was labeled differently; for example, ASH is a protonated state of ASP.

The structure of IC was obtained from Protein Data Bank (PDB code: 1CAR) (Arnott et al., 1974). Similar structures for KC and LC were derived from IC using Avogadro, an open-source molecular builder and visualization tool. Version 1.2.0n. http://avogadro.cc/. LMP and SA structures were obtained from PubChem with further adjustment into an appropriate length and charge with Avogadro (PubChem, n.d.-a, PubChem, n.d.-b). All polysaccharides were optimized using MMFF94s with default geometry optimization through Avogadro (Halgren, 1999; Hanwell et al., 2012). All polysaccharides and protein structures were prepared by merging non-polar hydrogen, adding Gasteiger charges based on electronegativity to obtain an appropriate electrostatic interaction, and fixing all torsions before docking with AutoDock tool 1.5.7 (Sanner, 1999).

The molecular docking was performed using blind docking since the binding site was not predetermined like the substrate using AutoDock Vina 1.2.3 software (Eberhardt et al., 2021). The grid dimension we used was 126 x 126 x 126 in the unit of Å with 1.000 Å spacing (Panda et al., 2020). The docking was visualized using Discovery Studio visualization software (BIOVIA, Dassault Systèmes, San Diego, v24.1.0) and ChimeraX (Pettersen et al., 2021).

### 2.7 Statistical analysis

The data were shown as mean values with standard deviation (SD) in triplicate (n = 3). A one-way analysis of variance (ANOVA) was done using GraphPad Prism10 (GraphPad Software Inc., US) and JMP Pro16 (SAS Institute, US). The differences between mean values were evaluated using the Tukey HSD comparison test with a p-value <0.05, indicating statistical significance. GraphPad Prism10 was also used to plot all the graphs.

## 3 Results and discussion

### 3.1 Optimization of coacervate complex

#### 3.1.1 Selection of candidates for anionic polysaccharides

**Fig. 1:**
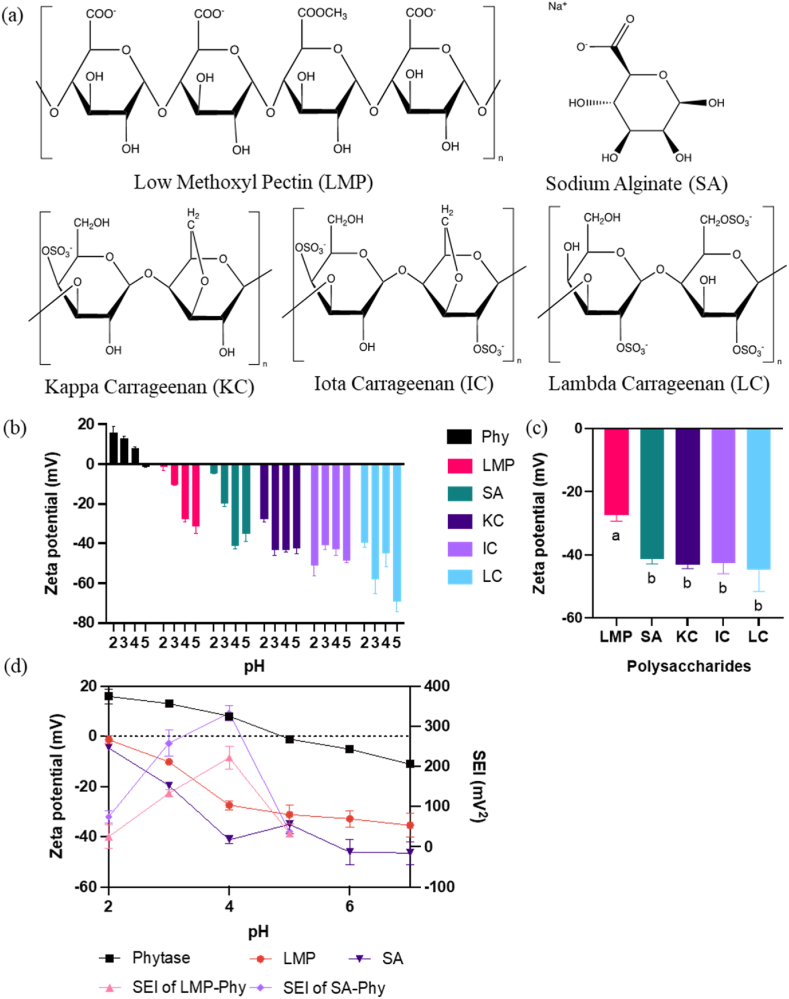
(a) Molecular structures of selected polysaccharides (PSs) created with ChemDraw, (b) zeta potential of phytase (Phy) (2 mg mL^−1^), and PSs (2 mg mL^−1^) including low methoxyl pectin (LMP), sodium alginate (SA), kappa-Carrageenan (KC), iota-Carrageenan (IC), and lambda-Carrageenan (LC), (c) zeta potential at pH 4 with Tukey's multiple comparison test (p < 0.05), and (d) zeta potential of LMP, SA and Phy with strength of electrostatic interaction (SEI) at different pH condition.

The five anionic polysaccharides commonly used in the food industry were classified into two groups based on functional groups: carboxyl and sulfate. The carboxyl group includes low methoxyl pectin (LMP) and sodium alginate (SA). The sulfate group includes κ-Carrageenan (KC), *ι*-carrageenan (IC), *λ*-carrageenan (LC), these carrageenans have varying charge densities (Fig. 1(a)). Above pH 2, carboxyl and sulfate functional groups make the surface of the polysaccharides negatively charged (Fig. 1(b)). We calculated the appropriate pH range using the strength of electrostatic interaction (SEI) by using an absolute zeta potential number for both the enzyme and the polysaccharide, then multiplying them together to get the SEI value (Huang et al., 2024; Lan et al., 2020; Yuan et al., 2017).

#### 3.1.2 Optimal pH condition

Coacervation relies on the surface charge of biopolymers to form a complex and is often influenced by pH level. The carrier materials, the polysaccharides (PSs), must have an opposite charge with the targeted enzyme to form a coacervate complex effectively. All PSs exhibited negative surface charge across all pH levels, while the carrageenans showed a slightly higher negative charge than the members of the carboxyl groups. Among the carrageenan types, lambda carrageenan exhibited the highest negative zeta potential due to its greater number of sulfate groups (Fig. 1(b)). Proteins such as enzymes have a unique isoelectric point that allows a change in surface charge at different pH levels. The suitable pH for complexation is where

phytase is positively charged; the isoelectric point of phytase is around pH 5, above which phytase would be negatively charged. Therefore, we focused on calculating the strength of the electrostatic interactions (SEI) at pH levels at or below pH 5 (Fig. 1(d)).

The solution's pH level impacts the enzyme's activity, such that suitable pH levels for maintaining phytase activity were found to be above pH 3 and below pH 7 (Fig. S3(a)), which agrees with literature reports (Naves et al., 2012). Therefore, the pH range that aligns with our

two criteria, a positively charged phytase for coacervation and high phytase activity, is between pH 3 and 4.

The strength of electrostatic interaction (SEI) is used to estimate the appropriate pH range for coacervate complex formation and indicates the magnitude of the attractive and repulsive forces(Lan et al., 2020). SEI indicated an attractive interaction for the carboxyl group with phytase at pH 4 and because the zeta potential for each carrageenan was not significantly different at a 95% confidence level from the zeta potential of sodium alginate (Fig. 1(c) and (d)). Thus, for comparison between carboxyl and sulfate groups, we used pH 4 as the optimum pH condition for coacervate formation.

#### 3.1.3 Optimal complexation ratio

To optimize the amount of enzyme in the coacervate and to measure how much phytase can be incorporated, we investigated different ratios of the enzyme to polysaccharides with the goal of achieving charge neutrality. The phytase-polysaccharide (Phy-PS) mixtures were prepared at pH 4 to optimize the complexation ratio, and for each ratio, the zeta potential, particle sizes, and turbidity were measured. All polysaccharides showed a high negative charge above −30 mV at pH 4, and phytase had a positive charge around 8 mV at pH 4 (Fig. 1(b)).

**Fig. 2:**
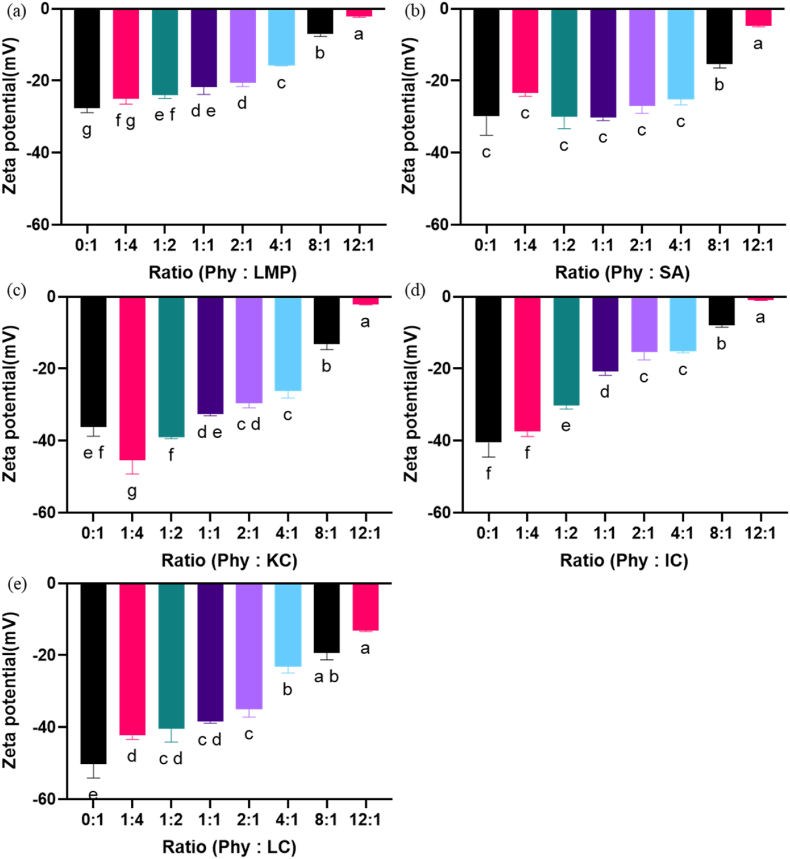
Zeta potential of complex mixture at pH 4 between phytase and (a) low methoxyl pectin (LMP), (b) sodium alginate (SA), (c) kappa-Carrageenan (KC), (d) iota-Carrageenan (IC), and (e) lambda-Carrageenan (LC) at different v/v ratio of Phy: Polysaccharides(PSs), including 0:1,1:4, 1:2,1:1, 2:1, 4:1, 8:1, and 12:1. Connecting letter plot was used at the significance difference level of p ≤ 0.05.

As the ratio of Phy increased in the mixture, the zeta potential gradually approached net zero charge for most PSs at a Phy-PS ratio of 12:1; however, due to the extra sulfate groups on LC, the Phy-LC ratio did not reach net zero even at the highest ratio of Phy to LC (Fig. 2). The closest zeta potential value to zero often suggests the highest complex formation potential due to the electrostatic interaction and subsequent charge naturalization between the protein and the polysaccharide, which causes phase separation and a turbid mixture (Lan et al., 2020; Lin et al., 2022). This complexation is attributed to the electrostatic interaction between positively charged phytase and negatively charged polysaccharides, which creates a dipole-dipole moment (Dannenberg et al., 1999). Across the chosen ratios, 12:1 was the closest to achieving charge neutrality.

**Fig. 3:**
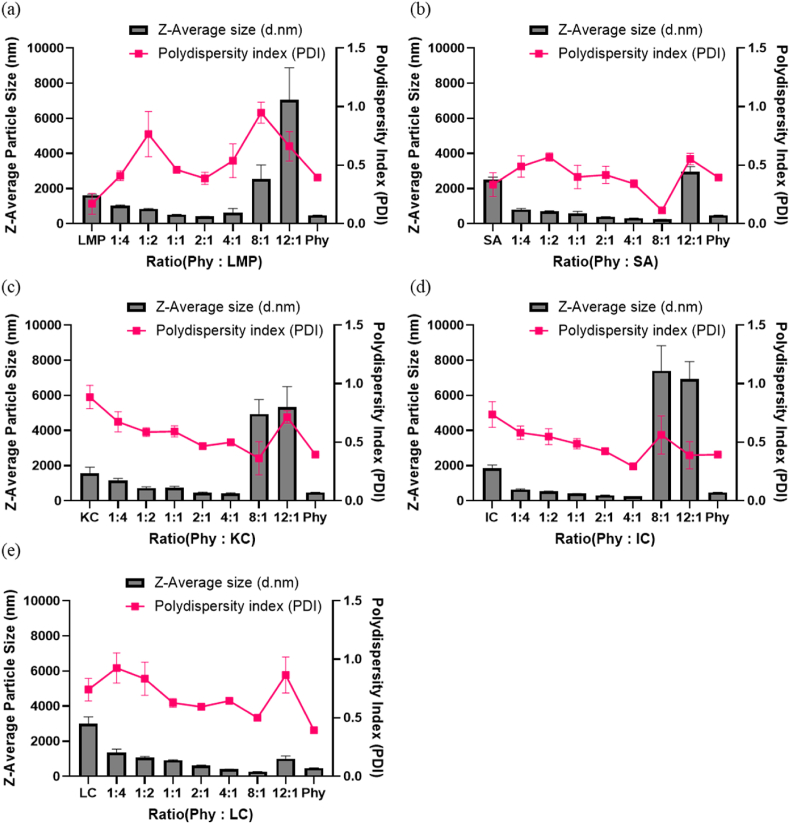
The particle size of complexes formed at different v/v ratios along with polydispersity index of phytase to polysaccharides; (a) low methoxyl pectin (LMP), (b) sodium alginate (SA), (c) kappa-Carrageenan (KC), (d) iota-Carrageenan (IC), and (e) lambda-Carrageenan (LC).

To further confirm the complex formation due to electrostatic interaction, we have measured particle size and turbidity of the polysaccharides alone, the phytase alone, and the complex mixtures at each ratio (Fig. 3&S2). Higher turbidity and particle size are associated with increased complex formation (Dong et al., 2024). All the complexes showed an increase in turbidity as we increased the amount of phytase. Particle size, however, decreased with increasing phytase content due to the enzymes' smaller size. For example, the coacervates with the 12:1 ratio of phytase to PSs had the largest particle size and highest turbidity, corresponding to the highest complex formation (Fig. S2).

For the carboxyl group, the phytase-low methoxy pectin (Phy-LMP) and phytase-sodium alginate (Phy-SA) complex mixtures had the highest average particle size at a ratio of 12:1 (Phy: PS). The size of the phytase, LMP, and SA particles before complexation were much smaller

compared to the particle size found at the 12:1 (Phy: PS) ratio, implying complexation between the phytase and polysaccharides (Fig. 3(a) and (b)).

The sulfate group, however, showed the largest particle sizes at 8:1 and 12:1 for Phy-KC and Phy-IC (Fig. 3(c) and (d)). This is partly due to the gelation caused by the crosslinking of the unique double helixes structure of KC and IC; thus, the complex does not require as much positive charge from phytase to form a complex as part of the complex formation was induced through hydrogen bonding via the unique structure (Yegappan et al., 2018). This phenomenon does not occur in LMP and SA because they prefer to gel in the presence of divalent cations such as Ca^2+^ and Fe^2+^ (E. Yang et al., 2024; X. Yang et al., 2018). LC did not for a gel because it does not have a double helix structure for crosslinking (Lam & Nickerson, 2014; Yegappan et al., 2018). Although the Phy-LC mixture complex at 12:1 had a smaller particle size and lower turbidity than other complexes, the Phy-LC mixture still showed an increase in size, indicating complex formation (Fig. 3(e)). This implied that the complex likely only formed through the electrostatic interaction, which required a higher phytase content than other PSs needed to achieve a phase separation (Obermeyer et al., 2016). Thus, we selected the Phy: PSs ratio of 12:1 for further experiments for phytase activity comparisons.

### 3.2 Immobilization efficiency, yield, and loading capacity of optimized complexes

Immobilization efficiency for the carboxyl group was highest for SA at ∼70% and close to 60% for LMP; the difference was not statistically significant at a 95% confidence level. Within the sulfate group, KC had the highest immobilization efficiency at 80%, and LC had the lowest at 70%; there was no statistical difference between members of this group either. A comparison of the two groups showed very little difference, though a comparison of LMP with.

**Fig. 4:**
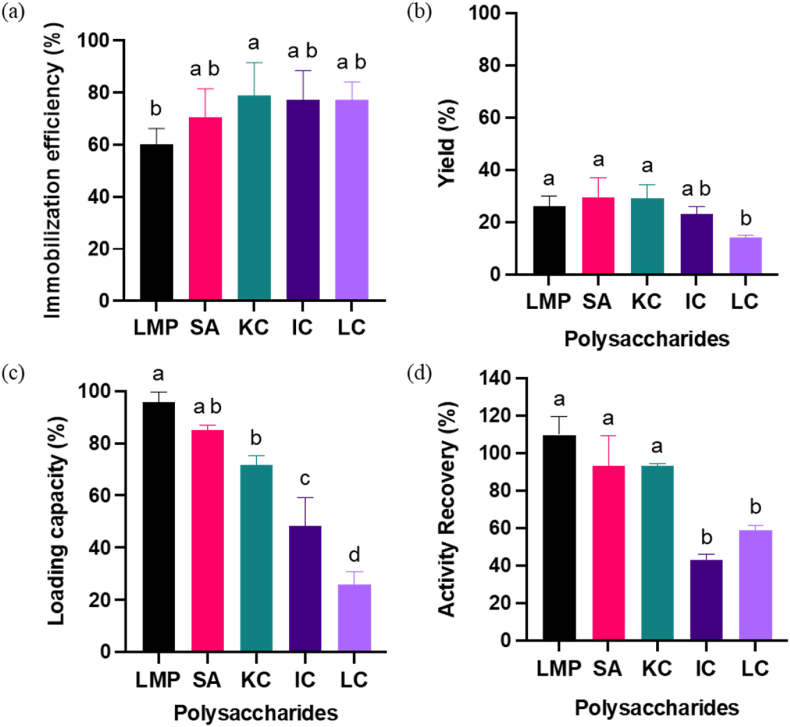
Coacervate complex of LMP, SA, KC, IC, and LC at 12:1 ratio; (a) % immobilization efficiency, (b) % yield, (c) %loading capacity, and (d) % activity recovery. A connecting letter plot was used at the significance difference level of p < 0.05.

KC was statistically different (Fig. 4(a)). A possible explanation is that LMP has a slightly lower zeta potential of ∼ −30 mV, while KC has ∼ −40 mV zeta potential at pH 4 (Fig. 1(c)). The significantly lower immobilization efficiency found in Phy-LMP complexes compared to Phy-KC complexes could be due to the difference in starting zeta potential since the intermolecular interaction may decrease when coacervation is a result of electrostatic interactions**.** This result implied that the higher negative charge on the polysaccharides resulted in a higher immobilization efficiency (McTigue & Perry, 2019).

The yield for the carboxyl group was highest for Phy-SA at ∼30% and approached 26% for Phy-LMP; the difference, however, was not statistically significant at a 95% confidence level. For the sulfate group, Phy-KC had the highest yield at ∼30%, and Phy-LC had the lowest at ∼14%. Only Phy-LC showed a statistically significant difference from Phy-KC and the members of the carboxyl group (Fig. 4(b)). Phy-LC showed the lowest yield, which we attribute to the lack of crosslinking; thus, less LC was captured or used in the immobilization of phytase.

For loading capacity, Phy-LMP was the highest for the carboxyl group at ∼96% and close to 85% for Phy-SA; the difference was not statistically significant at a 95% confidence level. There is a substantial difference within the sulfate group, while Phy-KC had the highest loading capacity at ∼72%. Phy-LC had the lowest at ∼26%. There was a statistical difference between the carboxylate and sulfate groups, although Phy-SA and Phy-KC were not statistically different (Fig. 4(c)). Loading capacity was influenced by molecular interactions induced by electrostatic and hydrogen bonding due to polysaccharide structure. The lowest loading capacity found in LC is attributed to a difference in binding conformation caused by its functional group charges and structure (Fig. S7).

The high immobilization efficiency and loading capacity are indicators of a good complex formulation because these properties minimize the loss of the targeted enzyme. With these quantifications, we conclude that Phy-SA and Phy-KC showed the best potential in forming a complex with phytase, followed by Phy-LMP. In addition to complex formation properties, enzyme activity recovery was used to confirm how the binding conformation was influenced by the polysaccharides'functional groups and the impact of the complex charge on enzyme activity.

### 3.3 Enzyme activity of optimized complexes

The recovery of enzyme activity did not significantly differ across the carboxyl group. In contrast, within the sulfate group, only the Phy-KC complex was significantly different from Phy-IC and Phy-LC at a 95% confidence level (Fig. 4(d)). The top three complexes, Phy-LMP, Phy-SA, and Phy-KC, were not significantly different in activity recovery. Using molecular docking, we have observed that these three conformations of Phy-LMP, Phy-SA and Phy-KC were different in comparison to Phy-IC and Phy-LC. The complexes showed a high activity recovery (Phy-LMP, Phy-SA and Phy-KC) have the best binding conformation outside of the active site, while the complexes that showed a lower activity recovery (Phy-IC and Phy-LC) have the best binding conformation around the active site area (Fig. S7). This is attributed to these polysaccharides' structure and strong negative charge, which make the most favorable binding site for IC and LC similar to the substrate binding site (phytate). In addition, Phy-LMP complexation further enhanced enzyme activity by approximately 10% in comparison with other complexes due to the electrostatic interactions that caused a specific protein-ligand conformation in which it increased the probability of collision between substrate and enzyme as similarly observed in the previous study in the coacervate complex between β-Glucuronidase and carboxy methyl cellulose (CMC) (Li et al., 2010) (Fig. S7). The observed conformation of Phy-LMP through molecular docking showed a distinct binding area in comparison to Phy-SA and Phy-KC (Fig. 7(g)–S7(a&d)). These results emphasize the importance of selecting types of polysaccharides for the best activity recovery and complex stability.

### 3.4 Characterization of optimized complex

#### 3.4.1 FTIR analysis

**Fig. 5:**
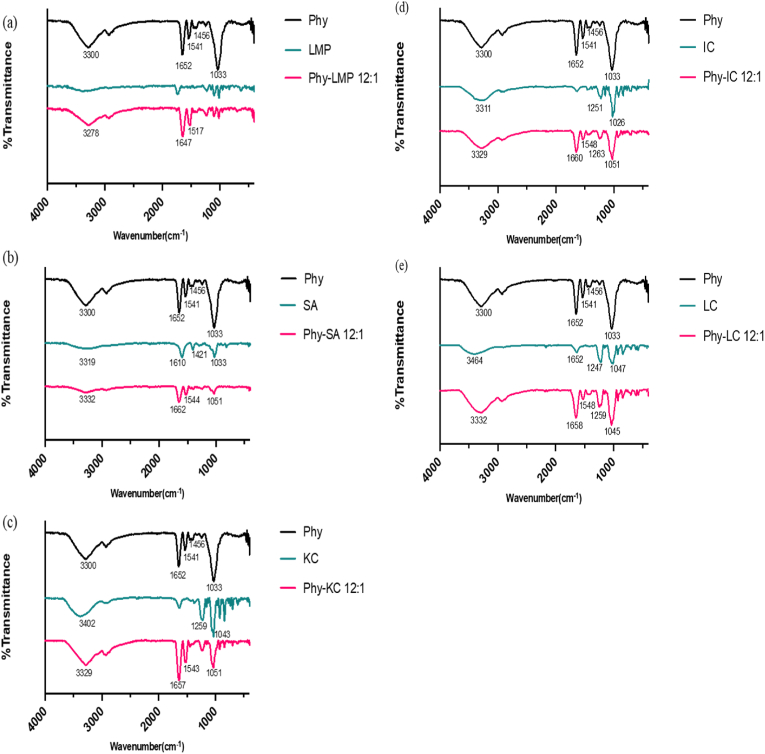
FTIR of (a–e) Phytase, phytase complexed with polysaccharides, and polysaccharides including LMP, SA, KC, IC, LC (respectively).

All complexes formed at a 12:1 ratio and pH 4 were evaluated using FTIR to confirm the interaction between phytase and the PSs (Fig. 5 and S5). In the phytase spectrum, the three bands at 3300 cm^−1^ indicated the O-H and N-H stretch, 1652 cm^−1^ and 1541 cm^−1^ indicated the presence of amide I and II, which are the C=O and N-H bands commonly found in proteins, and the band at 1033 cm^−1^ indicated the presence of a C-O stretch (Fig. 5(a)–(d)) (Andrade et al., 2019; Mashhadi Farahani et al., 2022). LMP and SA have a band in the region of 900–1200 cm^−1^ associated with the C-O stretch of the primary alcohol, and C=O (carbonyl) stretch around 1650 from the carboxyl group (More et al., 2024).

After complexation, the Phy-LMP and Phy-SA complexes showed the disappearance of the C-O stretch and a decrease in the intensity of the band associated with the O-H and N-H stretch from the phytase spectrum (Fig. 5(a)). This indicated that the interaction site between phytase and LMP or SA was mainly hydrogen bonding between the oxygen from the carboxyl group of LMP and SA and the hydrogen from the amine group of phytase (Sargolzaee et al., 2024). Uncomplexed, KC showed similar bands to phytase and a unique band indicating S=O stretch at the 1259 cm^−1^ band due to its sulfate group (Fig. 5(c)). The Phy-KC complex showed a decrease in the intensity of the S=O stretch and the C-O stretch, indicating the interaction site mainly occurred between the sulfate group of KC and the amino group of phytase involved hydrogen bonding (Tang et al., 2019). The FTIR spectra for Phy-IC and Phy-LC differed slightly from Phy-KC, with a band at 1548 cm^−1^ for amide II. The decrease in the band transmittance suggested that the decline in activity recovery found in Phy-IC and Phy-LC was likely due to the protein-polysaccharide conformation, specifically the interaction around the amide II of the protein (Alavi et al., 2018). To further confirm how the type of polysaccharide can influence the activity, we performed SDS-PAGE to assess the solubility of phytase in the solution after immobilization.

#### 3.4.2 SDS-PAGE determining phytase retention in complex

The complexes were analyzed using SDS-PAGE to determine protein solubility and quantity after dispersing in DI water (Bao et al., 2022; Brejnholt et al., 2011; Quan et al., 2004). Mild heat and a reducing agent were used to denature phytase in preparation for SDS PAGE. The detergent sodium dodecyl sulfate (SDS) was used to release the protein from the polysaccharides and cleave the disulfide bond to unfold the phytase into a linear chain with a negative charge (Al-Tubuly, 2000). The M lane indicated the molecular weight of the marker protein, while lanes 1–6 were phytase, Phy-LMP, Phy-SA, Phy-KC, Phy-IC, and Phy-LC, respectively (Fig. S6). Phytase had two strong bands at 15 and 20 kDa (Lane 1; Fig. S6). Complexes between Phytase and LMP/SA/KC showed similar strong bands with phytase itself, but Phy-IC and Phy-LC exhibited lower intensity bands. This difference suggested that the complex Phy-IC and Phy-LC may have strongly interacted with phytase, altered the protein's structure, and caused a slower phytase release after redispersion in DI water, which affected how much phytase presented for the binding of SDS to the protein backbone and resulted in lower intensity bands. This result aligned with the activity recovery data as we have seen less phytase activity in Phy-IC and Phy-LC. In this context, we conclude that phytase was successfully immobilized on the polysaccharides, and the intensity of the bands was influenced by the binding of IC and LC.

#### 3.4.3 SEM analysis

**Fig. 6:**
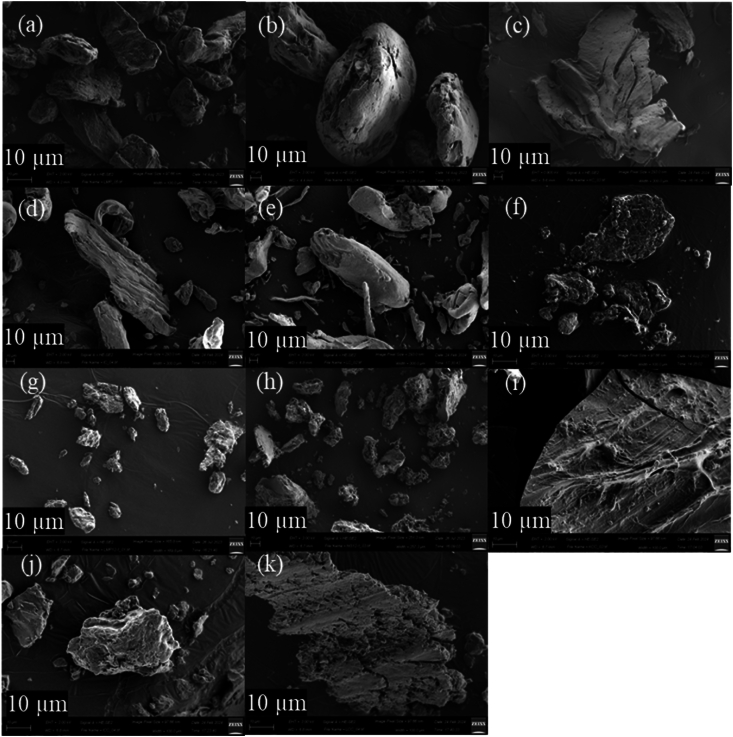
SEM image of anionic polysaccharides powder (a–e); LMP, SA, KC, IC, LC, respectively, (f) lyophilized powder of phytase, (g–k) and lyophilized powder of complex 12:1; Phy-LMP, Phy-SA, Phy-KC, Phy-IC, Phy-LC, respectively, at 10 μm scales.

SEM images (Fig. 6) were collected at 2, 10, and 20 μm magnifications to explore the surface morphology of the LMP, Phy-LMP complex, SA, and Phy-SA complex. The surface of the anionic polysaccharides LMP and SA appeared to be smooth with an irregular shape (Jong et al., 2023; Olechno et al., 2021). The phytase complexes of both polysaccharides (Fig. 6(a) and (b)) at the magnification of 2 μm revealed a rougher distribution throughout the particle, possibly indicating protein formation on the surface of anionic polysaccharide particles. The powder formed by coacervation can result in phase separation and, therefore, protein aggregated on the surface of polysaccharides as the size of the protein is much smaller than the polysaccharide. The SEM structure helped explain the Phy-PS complexes' high enzyme activity recovery, that is, the enzyme was mostly immobilized on the surface such that the active sites were still available for substrate interaction.

**Fig. 7:**
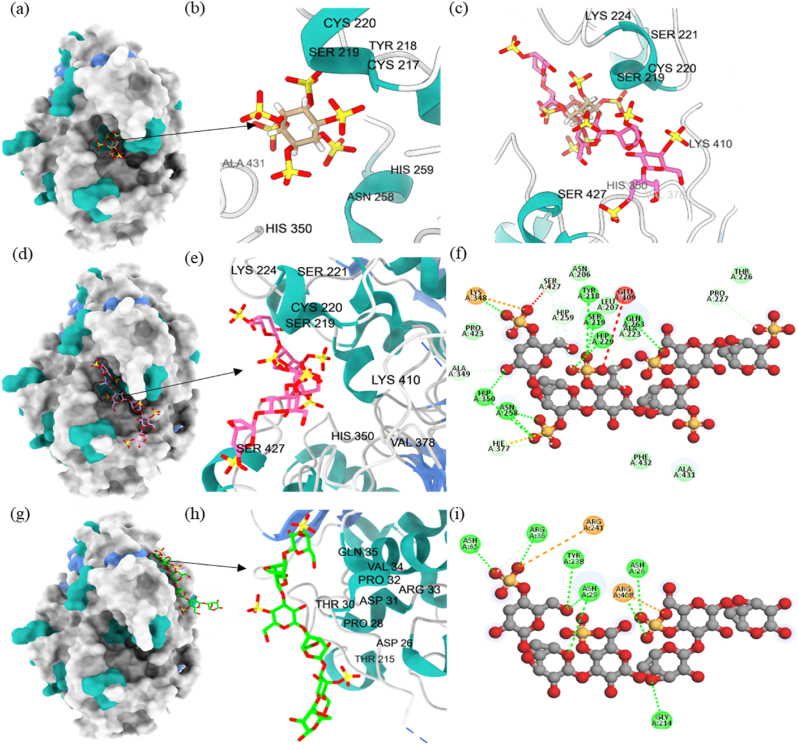
Molecular structure of (a–b) Phy-Phytate (6GJ2-substrate, labeled in cream), (c) docking conformation of Phy-IC (labeled in pink) with the substrate, (d–e) docking conformation of Phy-IC with the best binding affinity at −61 kJ/mol, (f) intermolecular interaction between Phy-IC, (g–h) docking conformation of Phy-KC (labeled in bright green) with the best binding affinity at −51 kJ/mol, (i) intermolecular interaction between Phy-KC. (a,d,g) The secondary structure of phytase was labeled by colors, in which grey dictated the coils, light sea green dictated the helixes, and cornflower blue dictated the strands. (f, i) The intermolecular interactions were also labeled in color, in which green colors represented hydrogen bonds, including van der Waals and C-H bonds, orange color represented an attractive charge, and red represented an unfavorable interaction, including charge repulsion and acceptor/donor clash.

#### 3.4.4 Molecular docking

Molecular docking is often used to predict the interaction between proteins and small ligands for drug discovery (Agu et al., 2023; Sahoo et al., 2022). It is rarely used for the prediction of larger molecules like polysaccharides due to the limitation in the structure database with a compatible structure format for docking software. Traditional docking methods may not consider the change in the external environment. To account for this, we chose an appropriately protonated state for the enzyme using Amber Force Field (Amber FF) to adjust to the coacervate formation pH (Jurrus et al., 2018). We simplified the length of polysaccharides to 6 repeating polysaccharide monomers for docking purposes. Although this simplification may decrease the complexity of the structure, it increases the flexibility of ligand conformation on the surface of phytase (Makshakova et al., 2021).

We have used the method of blind docking by building the grid box covering the entire phytase and obtained the best binding conformation with binding affinity and the intermolecular interaction between Phy-PSs through the ranking algorithm in AutoDock Vina 1.2.3. (Fig. 7, S7). Although positively charged amino acids were distributed randomly in phytase, molecular docking revealed that all Phy-PSs's favorable conformation is located at the alpha helix domain. A previous study on the molecular docking between 18 monomers of KC and lysozyme also showed a similar behavior in which the complex was stabilized by arginine and lysine on the alpha helix domain (Makshakova et al., 2021). The structure conformation also revealed that in the complex between Phy-IC and Phy-LC, the PSs bonded at the active site pocket where the substrate would bind to the enzyme. In contrast, the most favorable conformations were found for Phy-LMP, Phy-SA, and Phy-KC, which did not appear to be at the enzyme's active site. Therefore, the lower activity recovery we observed for Phy-IC and Phy-LC is most likely caused by the binding location of IC and LC to the phytase. Although the interaction between Phy and LC might be more robust due to stronger negative surface charges, the unfavorable conformation at the phytase active site caused a decrease in activity recovery. The intermolecular interaction results confirmed our hypothesis regarding the electrostatic interactions and hydrogen bonding as the primary intermolecular interactions (Fig. 7(f)–(i)).

## 4 Conclusions

Our study optimized the complex coacervation between a wheat-originated enzyme, phytase, and five negatively charged polysaccharides with different functional groups through the use of molecular docking and enzyme activity. A 12:1 enzyme-to-polysaccharides ratio at pH 4 yielded optimal results according to the zeta potential, turbidity, and particle size. Between carboxylate and sulfate groups, the polysaccharides with carboxylate functional groups showed better activity recovery, while the polysaccharides with sulfate functional groups showed a better immobilization efficiency. Among the coacervate complexes formed, the most effective complex was made with LMP. The Phy-LMP coacervate showed excellent activity recovery with good immobilization efficiency, loading capacity, and yield. We also confirmed the importance of the coacervate conformation through molecular docking. Coupled with our experimental results we could confirm that phytase activity decreased when the polysaccharides blocked the enzyme's active site. Thus, this shows the ability of molecular docking to serve as a tool to increase efficiency in selecting carriers to preserve enzyme activity.

One limitation of using molecular docking as a tool for screening appropriate polysaccharides is that it primarily focuses on electrostatic interactions. If the complex requires additional processes, such as crosslinking or heating, molecular docking may not be as efficient. This is because molecular docking does not take into account the water in the system, and these additional processes necessitate the solvent as a participating reagent. Future research should combine the use of molecular docking with molecular dynamic simulation to broaden the complex formation system. In addition, more investigation on enzyme stability could be further done with appropriate conditions depending on the application, including but not limited to thermal treatment, gastric digestion, ionic strength, etc.

## CRediT authorship contribution statement

**Waritsara Khongkomolsakul:** Writing – review & editing, Writing – original draft, Visualization, Investigation, Formal analysis, Conceptualization. **Eunhye Yang:** Writing – review & editing, Conceptualization. **Younas Dadmohammadi:** Writing – review & editing, Supervision, Conceptualization. **Hongmin Dong:** Writing – review & editing, Investigation. **Tiantian Lin:** Writing – review & editing, Methodology, Conceptualization. **Yunan Huang:** Writing – review & editing, Investigation. **Alireza Abbaspourrad:** Writing – review & editing, Supervision, Resources, Project administration, Funding acquisition, Conceptualization.

## Data availability statement

All data associated with this manuscript is available at: https://doi.org/10.5281/zenodo.14186386.

## Generative artificial intelligence (AI) statement

The authors certify that generative AI was not used in preparing this article. Non-generative AI, such as spelling and grammar checkers in Office 365 and Google Docs, and citation managing software, was used. All instances when non-generative AI was used were reviewed by the authors and editors.

## Declaration of competing interest

The authors declare that they have no known competing financial interests or personal relationships that could have appeared to influence the work reported in this paper.

## Data Availability

Data will be made available on request.
