# Supplementary material for: Enzyme immobilization with plant-based polysaccharides through complex coacervation
Source: Lebensm Wiss Technol. 2025 Mar 1;219:117537. doi: 10.1016/j.lwt.2025.117537 (PMC11867993; doi:10.1016/j.lwt.2025.117537)
Supplement: Multimedia component 1 [file mmc1.docx]

***Supplementary Material***

**Phytase Immobilization with Plant-Based Polysaccharides through Complex Coacervation**

Waritsara Khongkomolsakul, Eunhye Yang, Younas Dadmohammadi, Hongmin Dong^a^, Tiantian Lin, Yunan Huang, and Alireza Abbaspourrad*

Department of Food Science, College of Agriculture & Life Sciences, Cornell University, Stocking Hall, Ithaca, New York, 14853, United States

^*^Corresponding authors, Alireza Abbaspourrad (Alireza@cornell.edu)



**Figure S1.**  BCA standard curve of a concentration from 0-2 mg mL^–1^ of (a) phytase and (b) BSA.




**Figure S3.** Phytase activity (a) at pH 2-7 (2 mg mL^–1^) and (b) phytase standard curve measured at 820 nm using UV vis for calculating % activity recovery.

**

**

**Figure S4.** (a) Lyophilized powder at 12:1 of Phy-LMP, (b) Phy-SA, (c) Phy-KC, (d) Phy-IC, and (e) Phy-LC.



**Figure S5**. FTIR of (a) low methoxyl pectin (LMP), Phy-LMP complexes at different ratios, and phytase (Phy), and (b) kappa-Carrageenan (KC), Phy-KC complexes at different ratios, and phytase (Phy).



**Figure S6.** SDS-PAGE of phytase and complexes at 12:1 ratio. Lane M: Molecular weight markers. Lane 1: Phytase. Lane 2: complex Phy-LMP. Lane 3: complex Phy-SA. Lane 4: complex Phy-KC. Lane 5: complex Phy-IC. Lane 6: complex Phy-LC.



**Figure S7.** Molecular docking conformation of (a-b) Phy-LMP with the best binding affinity at -55 kJ/mol, (c) intermolecular interaction between Phy-LMP, (d-e) molecular docking conformation of Phy-SA with the best binding affinity at -55 kJ/mol, (f) intermolecular interaction between Phy-SA, (g-h) molecular docking conformation of Phy-LC with the best binding affinity at -59 kJ/mol, and (i) intermolecular interaction between Phy-LC. (a,d,g) The secondary structure of phytase was labeled by colors, in which the grey surface color dictated the coils, the light sea green color dictated the helixes, and the cornflower blue dictated the strands. (c, f, i) The intermolecular interactions were labeled in color between the amino acid and polysaccharide and green colors represented hydrogen bonds including Van der Waals and C-H bond, orange color represented attractive charge, and red represented an unfavorable interaction including charge repulsion and acceptor/donor clash.



**Figure S8.** Box plot showing the binding affinities in kilocalorie per mole for phytase with different polysaccharides (consisting of LMP, NG, KC, IC, and LC, respectively) with 95% confidence intervals (CI) and the outlier outside of 1.5 interquartile range, IQR was shown in black diamond.
